# A year in the life of the streptococcus

**DOI:** 10.1186/1546-0096-9-S1-P213

**Published:** 2011-09-14

**Authors:** ET Mosley, AM McMahon, F Shackley, C Waruiru

**Affiliations:** 1 Sheffield Children’s Hospital, Sheffield, UK; 2 Leeds Children’s Hospital ,Leeds, UK

## Background

Mild streptococcal infections infection are extremely common, however invasive infections can lead to high mortality rates. Described in 1982, post streptococcal reactive arthritis (PSRA) has been reported with increasing frequency.

## Aim

We aimed to look at the number of children presenting to a Tertiary Children’s Hospital, with serological significant streptococcal illnesses, diagnoses and antibiotic treatment of those patients.

## Method

Over a One year period of ASOT ( anti streptolysin O) results from a tertiary Children’s Hospital were reviewed. The ASOT results documented, paired data of ASOT & Anti DNase B (anti Deoxyribonuclease B antibodies) reviewed for correlation. Diagnoses were obtained using clinical notes.

Throat swabs results were reviewed where performed. The antibiotic treatment advised documented.

## Results

ASOT January 2009- December 2010 = 645

N° patients=359

N° ASOT’s >400 = 100 (28 %)

Notes reviewed = 95/100 patients

**Table:** 

Diagnosis by System	Number of patients
Rheumatology	18

Dermatology	18

ENT	17

Respiratory	12

Infectious diseases	11

Nephrology	8

Neurology	5

Chronic Fatigue	3

Gastroenterology	2

Cardiac	1

**Table:** 

Rheumatological Diagnosis	Number (18/95)
Juvenile idiopathic arthritis	4

Systemic Onset Juvenile Ideopathic arthritis	2

Post streptococcal Reactive Arthritis	3

Vasculitis	5

Kawasaki	2

Periodic Fever	2

Where both ASOT and Anti DNAse B were performed simultaneously (No =194), results correlated in 88%

Throat Swab performed in the group with ASOT >400 = 43/100

65 % patients with ASOT >400 were prescribed antibiotics

## Conclusions

Nearly 1/5 of patients with positive streptococcal serology had a rheumatological diagnosis, suggesting it is a significant trigger in rheumatological conditions.

This study highlights vigilance and alertness that patients with Streptococcal Infections may evolve or contribute to the development of a rheumatogical condition.

There is a need for a consensus opinion on treatment and eradication of streptococcal infection.

